# Outcome after intracranial hemorrhage under dabigatran and reversal with idarucizumab versus under vitamin-K-antagonists – the RIC-ICH study

**DOI:** 10.3389/fneur.2023.1212495

**Published:** 2023-07-24

**Authors:** Nils Kuklik, Anika Hüsing, Andreas Stang, Marcus Brinkmann, Christoph C. Eschenfelder, Christian Weimar, Hans-Christoph Diener

**Affiliations:** ^1^Institute for Medical Informatics, Biometry and Epidemiology, Medical Faculty, University of Duisburg-Essen, Essen, Germany; ^2^Centre for Clinical Trials Essen, University Hospital Essen, Essen, Germany; ^3^School of Public Health, Department of Epidemiology Boston University, Boston, MA, United States; ^4^Human Pharma Germany, Boehringer Ingelheim Pharma GmbH & Co. KG, Ingelheim, Germany; ^5^BDH Clinic Elzach, Elzach, Germany

**Keywords:** intracranial hemorrhage, anticoagulation, dabigatran, idarucizumab, vitamin-K-antagonists

## Abstract

**Background:**

Intracranial hemorrhage (ICH) is a rare but serious side effect associated with the use of oral anticoagulants, such as dabigatran. The specific reversal agent for dabigatran, idarucizumab, is available for the management of individuals with ICH. The aim of this study was to provide real-world evidence on patients with ICH and effective treatment with dabigatran and reversal with idarucizumab in clinical routine compared to those under effective treatment with vitamin-K-antagonist (VKA).

**Methods:**

Registration of Idarucizumab for Patients with IntraCranial Hemorrhage (RIC-ICH) is a non-interventional study conducted in 22 German stroke units that prospectively enrolled dabigatran patients treated with idarucizumab. Retrospective data from VKA patients served as reference population. Main objective was in-hospital mortality. Further objectives included change in bleeding volume, stroke severity, and functional status.

**Result:**

In-hospital mortality was 26.7% in 15 dabigatran and 27.3% in 88 VKA patients (hazard ratio 1.00, 95% CI 0.29–2.60). In patients with bleeding volume > 60 ml, mortality was lower in the dabigatran group (*N* = 6, 33%) compared to the VKA group (N = 15, 67%; HR 0.24, 95% CI 0.04–0.96). No differences were observed in secondary endpoints between dabigatran and VKA patients.

**Conclusion:**

These results, based on data from routine clinical practice, suggest that in-hospital mortality after idarucizumab treatment is comparable to that in patients pretreated with VKA. Due to the low precision of estimates, the results must be interpreted with caution.

## Introduction

Intracranial hemorrhage (ICH) is a rare but serious problem of oral anticoagulation including the non-vitamin-K-antagonizing oral anticoagulant dabigatran (1). Following the approval of idarucizumab in 2015, a specific reversal agent for dabigatran is available for the treatment of patients with serious bleeding complications or in need for urgent interventions or procedures (2). Although Idarucizumab has been shown to be effective and safe, more real-world data from prospective studies are important to investigate clinical endpoints after treatment with dabigatran and idarucizumab compared to other oral anticoagulants. The prospective, non-interventional Registration of Idarucizumab for Patients with IntraCranial Hemorrhage (RIC-ICH) was initiated to provide real-world evidence on important clinical outcomes. The main objective was to compare the in-hospital mortality in patients with ICH under effective treatment with dabigatran and treated with idarucizumab versus patients with ICH under effective vitamin-K antagonists (VKA) treatment.

## Methods

RIC-ICH is a non-interventional, multicenter study that prospectively recruited patients with clinically symptomatic ICH under effective treatment with dabigatran treated with idarucizumab in 22 German stroke units. Recruitment started in November 2019 with a planned inclusion of 95 dabigatran and 285 VKA patients and was terminated in December 2021. Because idarucizumab is generally used infrequently, dabigatran patients treated with idarucizumab were recruited prospectively to systematically enroll eligible patients in the stroke units and to minimize reporting bias. Also, it allowed systematic collection of information on adverse drug reactions related to idarucizumab treatment and reporting to the marketing authorization holder. Retrospective data from the most recent patients with ICH and effective treatment with VKA in the study centers during the study period served as reference population. Identical standardized clinical data collection forms were used for both the dabigatran and VKA patients. The study included real-world data on clinical routine until discharge from hospital or death. Main inclusion criteria were age ≥ 18 years, written informed consent (in dabigatran patients), primary intracranial hemorrhage as confirmed with CT, and effective anticoagulation treatment. For dabigatran patients who were not able to provide informed consent by themselves, this was given by a legal representative. In the case that it was not possible to obtain informed consent because of the medical condition of a patient without a legal representative, the investigator was authorized to decide on inclusion in the study and the appropriate consent was obtained as soon as possible. Ethical approval was given by the institutional review board of the University Duisburg-Essen (Reference number: 18-8517-BO). RIC-ICH has been registered with ClinicalTrials.gov (NCT04062097).

The main objective was to compare the intra-hospital mortality between dabigatran and VKA patients. Further comparisons included change in volume of ICH evaluated by CT 24-72 h after initial imaging, change in severity of stroke based on National Institutes of Health Stroke Scale (NIHSS) 72 h after hospital admission, functional status according to modified Rankin Scale (mRS) at discharge, as well as mortality after 7 and 30 days.

All endpoints were analyzed exploratively. Treatment effects were estimated as hazard ratios (HR) from Cox-proportional hazards regression analysis of mortality or relative risks (RR) from binary regressions, each with 95% confidence intervals.

## Results

In total, 15 patients treated with dabigatran and 88 patients treated with VKA were recruited. Mean age was 82.0 years (median 82) in the dabigatran group and 87.7 years (median 81) in the VKA group. Mean NIHSS on admission for dabigatran patients was 8.0 (VKA: 12.5) and mean mRS on admission was 3.5 (VKA: 4.1; see Table 1 for a summary of patients’ demographic and clinical characteristics). All dabigatran patients were treated with idarucizumab, and no adverse events causally related to reversal therapy were reported. Of 88 patients treated with VKA, 52 patients received treatment with prothrombin complex concentrate (PCC), 8 with vitamin K/PCC combination, 14 with vitamin K, and one with tranexamic acid.

**Table 1:** Patients’ demographic and clinical characteristics of the RIC-ICH study.

Characteristic	Dabigatran	VKA	Total
Total number of patients	15	88	103
Age, mean ± standard deviation (SD)	(N = 15) 82.00 ± 6.15	(N = 88) 78.68 ± 9.55	(N = 103) 79.17 ± 9.18
Sex:	(N = 15)	(N = 88)	(N = 103)
Male	8 (53.3%)	59 (67.0%)	67 (65.0%)
Female	7 (46.7%)	29 (33.0%)	36 (35.0%)
Body mass index, mean ± SD	(N = 13) 27.22 ± 4.17	(N = 43) 27.75 ± 5.77	(N = 56) 27.63 ± 5.41
Blood pressure systolic, mean ± SD	(N = 15) 157.60 ± 23.63	(N = 80) 167.73 ± 31.71	(N = 95) 166.13 ± 30.69
Blood pressure diastolic, mean ± SD	(N = 15) 84.20 ± 16.30	(N = 79) 91.68 ± 21.77	(N = 94) 90.49 ± 21.10
Smoking:	(N = 13)	(N = 38)	(N = 51)
Never smoker	10 (76.9%)	27 (71.1%)	37 (72.5%)
Current smoker	0 (0.0%)	4 (10.5%)	4 (7.8%)
Ex smoker	3 (23.1%)	7 (18.4%)	10 (19.6%)
NIHSS (admission), mean ± SD	(N = 15) 8.00 ± 6.28	(N = 77) 12.51 ± 9.93	(N = 92) 11.77 ± 9.55
Volume of intracranial bleeding in accordance to initial cerebral imaging (ml), mean ± SD	(N = 12) 51.12 ± 36.44	(N = 64) 41.41 ± 48.73	(N = 76) 42.95 ± 46.93
Modified Rankin Scale (premorbid):	(N = 15)	(N = 70)	(N = 85)
Grade 0	5 (33.3%)	28 (40.0%)	33 (38.8%)
Grade 1	0 (0.0%)	20 (28.6%)	20 (23.5%)
Grade 2	3 (20.0%)	9 (12.9%)	12 (14.1%)
Grade 3	3 (20.0%)	8 (11.4%)	11 (12.9%)
Grade 4	3 (20.0%)	4 (5.7%)	7 (8.2%)
Grade 5	1 (6.7%)	1 (1.4%)	2 (2.4%)
Modified Rankin Scale (admission):	(N = 14)	(N = 79)	(N = 93)
Grade 0	1 (7.1%)	1 (1.3%)	2 (2.2%)
Grade 1	0 (0.0%)	0 (0.0%)	0 (0.0%)
Grade 2	3 (21.4%)	11 (13.9%)	14 (15.1%)
Grade 3	1 (7.1%)	6 (7.6%)	7 (7.5%)
Grade 4	5 (35.7%)	22 (27.8%)	27 (29.0%)
Grade 5	4 (28.6%)	39 (49.4%)	43 (46.2%)
Concomitant Medication Nonsteroidal anti-inflammatory drug:	(N = 15)	(N = 88)	(N = 103)
Ibuprofen	1 (6.7%)	0 (0.0%)	1 (1.0%)
Other COX-1/COX-2 inhibitor	1 (6.7%)	0 (0.0%)	1 (1.0%)
Concomitant Medication Antiplatelets:	(N = 15)	(N = 88)	(N = 103)
Acetylsalicylic acid	0 (7.1%)	2 (2.3%)	2 (1,9%)
Clopidogrel	0 (0.0%)	1 (1.1%)	1 (1.0%)

In-hospital mortality was 26.7% (4/15) in the dabigatran group and 27.3% (24/88) in the VKA group (HR 1.00, 95% CI 0.29–2.60), with no differences when stratified by NIHSS on admission (see Table 2). Age stratification was uninformative due to sparse data. In patients with bleeding volume > 60 ml, mortality was different with 33% for the dabigatran group (2/6) and 67% in the VKA group (10/15, HR 0.24, 95% CI 0.04–0.96).

**Table 2:** Primary objective in-hospital mortality rates, RIC-ICH study.

Stratification	Mortality [%]		Mortality rate (deaths per 100 patient days)		Hazard ratio dabigatran versus VKA (reference) [95% CI]
	Dabigatran	VKA	Dabigatran	VKA	
None	26.7 (4/15)	27.3 (24/88)	2.8	1.5	1.00 [0.29; 2.60]
Age					
< 75 years	0.0 (0/2)	26.1 (6/23)	0.0	1.8	0.00 [NA; 6.78]
> = 75 years	30.8 (4/13)	27.7 (18/65)	3.0	1.5	1.10 [0.32; 2.96]
NIHSS on admission					
minor stroke (0–4)	16.7 (1/6)	4.5 (1/22)	2.0	0.4	5.83 [0.23; 150.70]
moderate stroke (5–15)	14.3 (1/7)	20.0 (7/35)	1.3	1.4	0.68 [0.04; 3.82]
severe stroke (>15)	100.0 (2/2)	60.0 (12/20)	13.3	8.5	1.53 [0.24; 5.86]
Size of initial hematoma					
0–29 ml	0.0 (0/5)	10.5 (4/38)	0.0	0.8	0.00 [NA; 4.96]
30–60 ml	0.0 (0/1)	36.4 (4/11)	0.0	5.8	0.00 [NA; 5.98]
> 60 ml	33.3 (2/6)	66.7 (10/15)	4.0	21.3	0.24 [0.04; 0.96]

All dabigatran patients included in volume change analysis (*N* = 10) showed a low hematoma growth of less than 6.5 ml or 33% between initial imaging and first control imaging compared to 77.5% (*N* = 31) of VKA patients (RR 1.29, 1.09–1.52). A subgroup of eight dabigatran patients (80%) showed an excellent outcome with hematoma growth of less than 20% (VKA: *N* = 27, 67.5%, RR 1.19, 0.81–1.73). Mean volume change in the dabigatran group was +2.2 ml and +4.0 ml in the VKA group.

Mean NIHSS after 72 h were 9.5 (dabigatran) and 11.0 (VKA), respectively. 23% of dabigatran and 18.2% of VKA patients showed worsening of stroke severity defined as increase of NIHSS ≥4 pts from admission until 72 h (RR 1.27, 0.41–3.97). In total, 46.7% of dabigatran patients and 24.4% of VKA patients showed a favorable outcome defined as mRS at discharge <= 3 (RR 1.91, 0.99–3.71).

Mortality after 7 days was 20.0% in the dabigatran group and 20.5% in the VKA group (HR 0.89, 0.21–2.62), and after 30 days 33.3% in the dabigatran group and 28.4% in the VKA group (HR 1.23, 0.41–2.98). See Table 3 for a summary of secondary objectives.

**Table 3:** Secondary objectives in terms of relative risk (RR), mean difference or hazard ratio (HR), RIC-ICH study.

Objective	Dabigatran	VKA	Comparing results after dabigatran vs. VKA (reference) [95% CI]
Hematoma growth below 6.5 ml or 33% from initial to first control imaging [% good or excellent outcome]	100.0 (10/10)	77.5 (31/40)	RR: 1.29 [1.09; 1.52]
Hematoma growth below 20% from initial to first control imaging [% excellent outcome]	80.0 (8/10)	67.5 (27/40)	RR: 1.19 [0.81; 1.73]
Mean change [ml] in hematoma volume [95% CI]	+2.2 [−4.1; 8.5]	+4.0 [−2.6; 10.7]	Mean difference: −1.8 [−10.6; 6.9]
Worsening of NIHSS by > = 4 points after 72 h [%]	23.1 (3/13)	18.2 (10/55)	RR: 1.27 [0.41; 3.97]
Favorable outcome at discharge (modified Rankin Scale 0–3) [%]	46.7 (7/15)	24.4 (20/82)	RR: 1.91 [0.99; 3.71]
Mortality after 7 days [%; deaths per 100 patient days]	20.0 (3/15); 3.3	20.5 (18/88); 3.8	HR 0.89 [0.21; 2.62]
Mortality after 30 days [%; deaths per 100 patient days]	33.3 (5/15); 3.4	28.4 (25/88); 2.5	HR 1.23 [0.41; 2.98]

## Discussion

Prior to the availability of idarucizumab, ICH in patients treated with dabigatran was associated with a mortality between 40 and 45% (3, 4). The use of idarucizumab markedly improved patient outcome, although mortality remained a critical concern as summarized in a meta-analysis from 2021 (overall mortality 18%, ranging from 2 to 40% between studies) (5). RIC-ICH was designed to supplement the limited availability of prospective observational studies and to provide real-world data reflecting the treatment in daily practice in patients suffering from ICH under treatment with idarucizumab.

Our data show that in-hospital mortality is similar between the dabigatran group and the VKA group, and is within the range of other prospective non-interventional studies on idarucizumab treatment in emergency bleeding situations (17.8% Yasaka et al., 29.4% Küpper et al.) (6, 7). The largest German cohort to date of patients with intracranial hemorrhage and idarucizumab reversal therapy had an in-hospital mortality of 15% in 40 retrospective cases (8). The open-label RE-VERSE AD study reported an in-hospital mortality of 18.8% (intracranial hemorrhage and gastrointestinal bleeding combined) (2). Different study designs and patient collectives as well as the relatively low precision of estimates in this study hamper direct comparisons and may be relevant reasons for differences in mortality and other outcomes within studies.

The subgroup of patients with a bleeding volume > 60 ml showed lower in-hospital mortality in dabigatran patients. Reversal of dabigatran with idarucizumab was associated with a lower mean increase in hematoma volume and an overall higher percentage of patients with good or excellent hematoma growth outcomes compared with VKA patients. Effective control of hematoma growth is particular important for patients with high initial hematoma volume and therefore may contribute to lower in-hospital mortality in dabigatran patients compared with VKA patients in this subgroup.

Since this study follows an observational real-world approach, as few exclusion criteria as possible were defined. Therefore, the results of this study are not generalizable to a specific VKA reversal therapy. In general, in view of a smaller than planned study cohort, the small number of patients included led to low precision estimates and limited the use of multivariable models to adjust for relevant patient characteristics and reduce potential bias. Therefore, conclusions drawn in this paper must be interpreted with caution, but provide an important basis for further larger observational studies.

This study was extremely difficult to conduct. The 22 participating hospitals were both privately and publicly owned hospitals, as well as university hospitals, spread across the main urban and rural areas in Germany. A dramatic decrease of the prescription of dabigatran therapy in the catchment area of participating hospitals was not anticipated. In Germany, the use of direct oral anticoagulants turned into a Factor-Xa-inhibitor dominated market, where dabigatran (Factor-IIa-inhibitor) took the last position during the past years. During the conduct of the study, as mentioned earlier, the number of patients on dabigatran treatment remained low in Germany. Adding the low frequency of major bleeding complications for dabigatran, especially ICH, lead to the low usage of the dabigatran-specific antidote idarucizumab. Certainly, there is no issue with idarucizumab, neither on the mode of action level, nor on the marketed product level (Praxbind®). The dabigatran-specific antidote idarucizumab is produced and available in many regions around the globe. Since its market introduction in 2015 the benefit/risk profile of idarucizumab remained remarkably positive, as also demonstrated by the results of this study. Thus, dabigatran remains an important treatment option globally, highlighting the relevance of the availability of further studies.

## Conclusion

In this prospective real-world study, in-hospital mortality after idarucizumab therapy in patients pretreated with dabigatran who experienced ICH was comparable to that in patients pretreated with VKA. The observation that specific therapy with idarucizumab achieves a similar mortality rate as treatment with prothrombin complex for bleeding in patients treated with VKA is reassuring. In view of the low number of patients and low precision of estimates, our results must be interpreted with caution.

## Data availability statement

The original contributions presented in the study are included in the article/supplementary material, further inquiries can be directed to the corresponding author.

## Ethics statement

The studies involving human participants were reviewed and approved by Institutional review board of the University Duisburg-Essen (Reference number: 18-8517-BO). The patients/participants provided their written informed consent to participate in this study.

## Author contributions

H-CD, CW, and MB designed the study. H-CD and CW supervised the project. NK administered the project, performed data curation, and drafted the original manuscript. AH performed formal analysis. All authors contributed to the article and approved the submitted version.

## Funding

This study received funding from Boehringer Ingelheim. The funder was not involved in the study design, collection, analysis, interpretation of data, the writing of this article or the decision to submit it for publication. All authors declare no other competing interests.

## Conflict of interest

H-CD has received honoraria for participation in clinical trials, contribution to advisory boards or oral presentations from: Abbott, BMS, Boehringer Ingelheim, Daiichi-Sankyo, Novo-Nordisk, Pfizer, Portola and WebMD Global. Boehringer Ingelheim provided financial support for research projects. H-CD received research grants from the German Research Council (DFG) and German Ministry of Education and Research (BMBF). H-CD serves as editor of Neurologie up2date, Info Neurologie & Psychiatrie and Arzneimitteltherapie, as co-editor of Cephalalgia, and on the editorial board of Lancet Neurology and Drugs. CE is employed by Boehringer Ingelheim.

The remaining authors declare that the research was conducted in the absence of any commercial or financial relationships that could be construed as a potential conflict of interest.

## Publisher’s note

All claims expressed in this article are solely those of the authors and do not necessarily represent those of their affiliated organizations, or those of the publisher, the editors and the reviewers. Any product that may be evaluated in this article, or claim that may be made by its manufacturer, is not guaranteed or endorsed by the publisher.
